# Dating app-facilitated infidelity, sexual attitudes, and personality

**DOI:** 10.3389/fpsyg.2026.1782214

**Published:** 2026-04-23

**Authors:** Lennart Freyth

**Affiliations:** 1 Psychology Department, University of Applied Sciences Döpfer, Cologne, Germany; 2 Behavioral and Social Sciences Institute, Vienna, Austria

**Keywords:** cheating, dark triad, dating apps, infidelity, sexual compulsivity, sexual risk behavior, sexuality, sexually transmitted infections (STI)

## Abstract

This study examined individual differences in sexual behaviors and attitudes (dating app sex, condom use, contraception responsibility, drinking on dates) among single and partnered dating app users (*N* = 495), alongside personality (i.e., narcissism, Machiavellianism, psychopathy, and sexual compulsivity) and sexual satisfaction. Analyses were stratified by sex to capture different sexual strategies. Dating app users were higher in sexual compulsivity than non-users. Sex among app users was more likely among users in committed relationships, confirming the prejudice of less trustworthy dating app users. Both sexes showed uncommitted sexual behaviors, but women displayed a more complex array of sexual strategies: Male users reporting sex were more Machiavellian and sexually compulsive than those who reported no sex with other users. Machiavellian women and those who were sexually satisfied were more likely to cheat via dating apps. Psychopathic men and narcissistic women preferred to drink during dates. The results are in line with previous intercultural research on infidelity and sexually transmitted infections, indicating that lifestyle and personality between individuals are of greater importance for public sexual health than differences between countries. Finally, potential implications of borderline behavior among dating app users are discussed.

## Introduction

Individual dating app use can better be predicted by the non-clinical Dark Triad traits of narcissism, Machiavellianism, and psychopathy compared to the Big Five traits (Freyth and Batinic, 2021). These traits are everyday quantitative differences between individuals (i.e., continuum principle, not qualitative differences as in clinical psychology) and share a focus on reproductive options (i.e., life history theory; see framework: Freyth et al., 2027). Each trait is linked to distinct fast-life history strategies (Jones and de Roos, 2017). On these apps, narcissists are perceived as charming and picked by others (Freyth and Batinic, 2021). Machiavellians search for status and dating options, which do not lead to a relationship; dissolution may be because of their strategic character (Weiser et al., 2018; Lyons et al., 2022). Psychopaths are thrill-seeking, impulsive, and hook up opportunistically. Users with elevated dark traits search (Lyons et al., 2022) and report more casual sex with other users (henceforth Tinder-sex; Freyth and Jonason, 2025). These individuals engage in infidelity offline (March et al., 2024), yet if they not only look for (Lyons et al., 2022) but also engage in dating app-facilitated cheating is still unknown. Other relevant but regularly overlooked aspects related to risky sexual behaviors (i.e., hook-ups, numerous partners, and unprotected sex; Flesia et al., 2021) are contraception and health. Therefore, this study extends prior knowledge about dating app users’ behaviors, attitudes (i.e., Tinder-sex; condom use, contraceptive responsibility, and alcohol on dates), traits (i.e., narcissism, Machiavellianism, psychopathy, and sexual compulsivity), and sexual satisfaction (i.e., ego-satisfaction and partner satisfaction) to single and partnered individuals.

Labeling dating app users as less trustworthy (Silva et al., 2019) is probably based on the short-term mating orientation of individuals and their intentions to cheat on these apps (Weiser et al., 2018). Sexually compulsive (i.e., lack of sexual impulse control and sexual preoccupation) individuals using Tinder (Shapiro et al., 2017) report more sexual partners in general and outside of committed relationships (Stupiansky et al., 2009), yet until today, associations with Tinder-sex are unexplored. It is unknown how these behaviors manifest differently relative to relationship status.

*H1*: Machiavellians and psychopaths cheat via dating apps.

Based on the different reproductive costs of sex, men and women have developed distinct preferences and courtship behaviors for finding intimate partners, especially short-term ones (Bode and Kushnick, 2021; Trivers, 1972): Men are more open to uncommitted sex, whereas women are more cautious when picking a partner (Haselton and Buss, 2000). Like smartphone apps, these sex differences appear universal (Schmitt, 2005). So, extended messaging on dating apps may create perceived security for women, facilitating Tinder-sex. Users engaged in more risky sexual behaviors elevate sexually transmitted infection (STI) risks (Flesia et al., 2021), especially when drinking (Lewis et al., 2012). Then, to avoid STIs, both partners must be healthy; while condoms reduce some risks, sexual infidelity increases risks. Ultimately, only contraception prevents unintended pregnancy.

*H2*: Women with a preference for casual sex (i.e., high narcissism, Machiavellianism, psychopathy, and sexual compulsivity) show the same risky behaviors as men (Mikach and Bailey, 1999).

Besides individual differences, countries and cultures differ in risk-taking and STIs. As STI rates in poorer countries are linked to the birth rate and less to socio-economic factors, differences within regions differ more strongly. For instance, in Europe between 1990 and 2019, STI rates in Norway, Spain, and Italy increased, while Germany reported decreasing rates (Zheng et al., 2022). Although this study explored individual differences within one country, future research might further explore intercultural differences, as there is still no consensus on how to describe the lacking pattern.

## Methods

### Participants and procedure

To detect differences (*f* = 0.25, *α* = 0.05) with a power of 0.95, samples of 192 (men and women) were needed. Data were collected using an online questionnaire via Respondi, a German-speaking panel (2.50€ incentive). The sample consisted of 495 respondents (288 men, 207 women) aged 16 to 70 (*M* = 41.99, *SD* = 14.18) after removing 57 participants (>2 missing items; response pattern analysis; average time per item below 1 s). Participants consented twice, once to the tracking panel and once to the survey before the start. After participants completed the survey, they were debriefed and thanked. Ethical clearance for the study was obtained from the ethics committee of the Psychological Institute of Johannes Kepler University Linz. The committee also recommended collecting the data at once to reach a larger sample size for this (focus on infidelity and health risks) and another study for statistical and economic reasons (Freyth and Jonason, 2023; psychometric comparison of shared variances of disagreeableness, the dark core, and the Dark Triad traits with sociosexuality facets; https://osf.io/keprj/overview?view_only=f4889709ed1b4f8fb97ce34eb6ade955).

### Measures

Participants were asked if they were currently in a committed relationship (*yes/no*), if they would be open to a new romantic partner (*yes/no*), open to a sexual adventure (*yes*/*no*), considered condoms a must on a date with a new acquaintance (*yes*/*no*), considered themselves in charge of contraception (*yes*/*no*), and if they considered alcohol part of a date (*yes*/*no*). If participants reported to the filter question that they “ever went on a date with someone they met on dating applications” they were asked: “how many sexual partners did you have organized via dating applications?.” This way, nobody reported Tinder-sex without being on a date. We transformed Tinder-sex into a binary variable (yes ≥ 1/no = 0) because of positively skewed data for reported partners, particularly men (Table 1).

**Table 1:** Descriptives and distribution of demographic variables (frequencies for binary variables).

	Men			Women			*t*-tests	
	*M(SD)*	*Skewness*	*Kurtosis*	*M(SD)*	*Skewness*	*Kurtosis*	*t*	*d*
Age	45.03 (14.97)	0.26	−0.69	40.86	0.24	−1.21	2.81*	0.27
Tindersex	10.63 (34.15)	5.94	42.25	3.54(6.64)	3.84	19.53	1.82	0.26
Narcissism	3.49 (1.46)	0.12	−0.66	3.56(1.53)	0.09	−0.77	−0.46	−0.04
Machiavellianism	2.97 (1.40)	0.55	−0.39	2.80(1.40)	0.49	−0.57	1.35	0.12
Psychopathy	2.92 (1.22)	0.68	0.44	2.48(1.22)	1.02	1.18	3.95**	0.36
Sexual compulsivity	1.82 (0.57)	1.59	3.32	1.55(0.47)	0.67	−0.17	4.94**	0.50
Sexual satisfaction (self)	3.57 (1.01)	−0.87	0.48	3.48(1.10)	−0.68	−0.15	1.01	0.09
Sexual satisfaction (partner)	3.21 (1.10)	−0.41	−0.43	3.19(1.13)	−0.33	−0.67	0.20	0.02

	*Frequency*	
Partnered	60%	55%
Open for a new partner	49%	42%
Open for casual partner (men)	38%	13%
Pro condom use	67%	57%
Contraception responsibility	65%	65%
Alkohol on a date	66%	70%
Tindersex (yes)	58%	66%
Tindersex and partnered (yes)	75%	70%

*d* = Cohen’s *d*.

*p < 0.05 and **p < 0.001.

The German Naughty Nine Scale (9 items, Küfner et al., 2015) was used. Participants reported how much they agree (1 = *I totally disagree*, 5 = *totally agree*; “I tend to….”) with statements assessing narcissism (e.g., “…seek prestige or status”; *ω* = 0.83), Machiavellianism (e.g., “…manipulate others to get my way”; *ω* = 0.80), and psychopathy (e.g., “…be callous or insensitive,” *ω* = 0.63). Items were averaged into indexes.

The German version of the Sexual Compulsivity Scale (10 items; Kalichman and Rompa, 1995) was used. Participants’ agreement (1 = *not at all like me*, 4 = *very much like me*) on sexual compulsivity (e.g., “I think about sex more than I would like to”) was captured. Items were averaged into a trait score (*ω* = 0.88).

The new sexual satisfaction scale short version in German (12 items; Štulhofer et al., 2010) was used. It captures participants’ satisfaction (1 = *not at all satisfied*, 5 = *extremely satisfied*) with ego-focused (e.g., “My body’s sexual functioning,” *ω* = 0.93) and partner/activity-focused sexual satisfaction (e.g., “The frequency of my sexual activity,” *ω* = 0.93). Items were averaged into indexes.

### Analysis

Men and women were analyzed separately because of their sex-specific sexual strategies. A 2 (relationship: yes/no) × 2 (dating app use: yes/no) × 2 (variable of interest: yes/no) ANOVA design was used as the default to explore individual differences in relationship status, dating apps use, Tinder-sex, condom use, and the responsibility for contraception with traits and sexual satisfaction as dependent variables (Table 2). If the group sizes of dating app users were below 10, a 2 × 2 design without dating app use was used (Bonferroni-corrected α = *0*.01; tendencies up to α = 0.05 are reported).

**Table 2:** Series of ANOVAs, reporting descriptive statistics for relationship status (relationship: yes/no) and risky sex styles.

	*Men*				*Women*			
	*M* (*SD*)		*t*	*d*	*M* (*SD*)		*t*	*d*
	*Condition = Yes*	*Condition = No*			*Condition = Yes*	*Condition = No*		
*Relationship*								
Narcissism	3.57 (1.49)	3.42(1.44)	0.82	0.10	3.48(1.40)	3.59 (1.66)	−0.49	−0.07
Machiavellianism	3.06 (1.45)	2.88(1.34)	1.08	0.13	2.74(1.37)	2.77 (1.36)	−0.18	−0.03
Psychopathy	3.09 (1.26)	2.73(1.14)	2.55*	0.30	2.48(1.20)	2.45 (1.26)	0.22	0.03
Sexual compulsivity	1.82 (0.60)	1.83(0.54)	−0.01	<0.01	1.56(0.49)	1.56 (0.46)	−0.07	−0.01
Ego-satisfaction	3.81 (0.84)	3.30(1.14)	4.31**	0.52	3.55(1.09)	3.34 (1.12)	1.35	0.20
Partner-satisfaction	3.46 (1.00)	2.91(1.16)	4.27**	0.51	3.37(1.09)	2.89 (1.13)	2.94*	0.43
*Dating app use*								
Narcissism	3.88 (1.47)	3.10 (1.36)	4.57**	0.54	3.74 (1.60)	3.36 (1.43)	1.80	025
Machiavellianism	3.23 (1.44)	2.71 (1.31)	3.19*	0.38	2.95 (1.46)	2.62 (1.28)	1.73	0.24
Psychopathy	3.08 (1.30)	2.77 (1.11)	2.14*	0.25	2.58 (1.33)	2.38 (1.11)	1.18	0.17
Sexual compulsivity	2.00 (0.59)	1.64 (0.49)	5.60**	0.66	1.66 (0.53)	1.46 (0.39)	3.16*	0.44
Ego-satisfaction	3.65 (0.99)	3.50 (1.05)	1.20	0.14	3.51 (1.11)	3.44 (1.11)	0.42	0.06
Partner-satisfaction	3.20 (1.11)	3.23 (1.10)	−0.21	−0.03	3.13 (1.18)	3.24 (1.09)	−0.67	−0.10
*Sex on tinder date*								
Narcissism	4.08 (1.37)	3.58 (1.62)	1.86	0.34	3.81 (1.54)	3.86 (1.87)	−0.12	−0.03
Machiavellianism	3.46 (1.33)	2.81 (1.51)	2.52*	0.46	3.00 (1.54)	2.94 (1.41)	0.18	0.04
Psychopathy	3.23 (1.28)	2.84 (1.26)	1.68	0.31	2.41 (1.29)	2.85 (1.40)	−1.45	−0.33
Sexual compulsivity	2.09 (0.57)	1.89 (0.56)	1.94	0.36	1.69 (0.54)	1.68 (0.57)	0.10	0.02
Ego-satisfaction	3.72 (0.83)	3.60 (1.17)	0.67	0.12	3.76 (1.09)	3.15 (1.19)	2.34*	0.54
Partner-satisfaction	3.33 (1.02)	3.03 (1.21)	1.46	0.27	3.40 (1.17)	2.64 (1.20)	2.83*	0.65
*Condom use*								
Narcissism	3.57 (1.44)	3.43 (1.50)	0.79	0.10	3.65 (1.57)	3.32 (1.43)	1.43	−0.08
Machiavellianism	3.00 (1.40)	2.95 (1.41)	0.30	0.04	2.85 (1.37)	2.61 (1.38)	1.17	0.18
Psychopathy	2.99 (1.25)	2.86 (1.18)	0.92	0.11	2.58 (1.29)	2.31 (1.05)	1.46	0.22
Sexual compulsivity	1.83 (0.60)	1.83 (0.60)	−0.09	−0.01	1.56 (0.49)	1.54 (0.45)	0.36	0.06
Ego-satisfaction	3.68 (0.94)	3.44 (1.11)	1.97*	0.24	3.45 (1.11)	3.55 (1.10)	−0.58	−0.09
Partner-satisfaction	3.31 (1.00)	3.09 (1.22)	1.72	0.21	3.16 (1.23)	3.26 (1.16)	−0.62	−0.09
*Contraception*								
Narcissism	3.49 (1.51)	3.53 (1.40)	−0.18	−0.02	3.56 (1.53)	3.51 (1.52)	0.19	0.03
Machiavellianism	2.87 (1.38)	3.19 (1.43)	−1.82	−0.23	2.84 (1.39)	2.66 (1.35)	0.22	0.04
Psychopathy	2.86 (1.26)	3.06 (1.13)	−1.30	−0.16	2.66 (1.35)	2.49 (1.24)	−0.59	−0.10
Sexual compulsivity	1.77 (0.52)	1.94 (0.65)	−2.39*	−0.30	1.54 (0.49)	1.59 (0.44)	−0.55	−0.09
Ego-satisfaction	3.69 (0.94)	3.38 (1.12)	2.47*	0.31	3.49 (1.07)	3.45 (1.18)	1.27	0.21
Partner-satisfaction	3.33 (1.02)	3.00 (1.22)	2.42*	0.30	3.21 (1.05)	3.13 (1.28)	1.98*	0.33
*Alcohol*								
Narcissism	4.27 (1.46)	3.68 (1.49)	2.31*	0.40	4.19 (1.64)	3.38 (1.57)	2.30*	0.50
Machiavellianism	3.46 (1.53)	3.17 (1.40)	1.13	0.20	3.26 (1.55)	2.72 (1.35)	1.71	0.37
Psychopathy	3.42 (1.35)	2.88 (1.24)	2.41*	0.42	2.70 (1.38)	2.47 (1.37)	0.77	0.17
Sexual compulsivity	2.09 (0.58)	1.98 (0.58)	1.11	0.19	1.71 (0.59)	1.66 (0.50)	0.43	0.09
Ego-satisfaction	3.70 (0.88)	3.69 (1.02)	0.10	−0.02	3.45 (1.18)	3.53 (1.16)	−0.29	−0.06
Partner-satisfaction	3.36 (1.07)	3.15 (1.12)	1.13	−0.14	3.10 (1.19)	3.07 (1.24)	0.11	0.02

*d* = Cohen’s *d*.

*p < 0.05 and **p < 0.001.

## Results

In this sample, men were older, more psychopathic, and sexually compulsive than women. Relatively, more partnered than single male Tinder users had Tinder-sex (*χ*^2^ = 25.26, *p* < 0.01, *Φ* = 0.30). Among women, more partnered than single women had Tinder-sex (*χ*^2^ = 37.06, *p* < 0.01, Φ = 0.43). The first series of 2 (single vs. non-single) × 2 (Tinder-sex: yes/no) ANOVAs focusing on Tinder-sex was run with traits and sexual satisfaction as dependent variables for men and women. No interactional effects were found for men (*F*[6, 126] = 0.98, *p* = 0.44, *η_p_*^2^ = 0.02). Men who had sex via dating apps were slightly higher in Machiavellianism (*F*[2, 130] = 5.67, *p* = 0.02 *η_p_*^2^ = 0.06) and sexual compulsivity (*F*[2, 130] = 5.61, *p* = 0.01, *η_p_*^2^ = 0.12). Women who were in a relationship and who had Tinder-sex were slightly more Machiavellian than women who had no Tinder-sex, while women who were single and had Tinder-sex were slightly less Machiavellians than those who had no Tinder-sex (*F*[2, 194] = 3.13, *p* < 0.05, *η_p_*^2^ = 0.03). Single women who had Tinder-sex were less psychopathic than those who had no Tinder-sex, with no difference between partnered women who had Tinder-sex or did not (*F*[2, 194] = 3.12, *p* < 0.05, *η_p_*^2^ = 0.03). Women having Tinder-sex, compared to those who had not, were slightly more satisfied with their own (*F*[2, 73] = 5.59, *p =* 0.04, *η_p_*^2^ = 0.02) and their partner’s sexual potential (*F*[2, 73] = 5.86, *p =* 0.02, *η_p_*^2^ = 0.03).

Main effects (Table 2) indicated that partnered men were more satisfied with their own sexual experiences and their partner’s sexual activity than single men. Men using dating apps were more narcissistic, Machiavellian (slightly), psychopathic (slightly), and sexually compulsive. Partnered women were more satisfied than their single counterparts. Women using dating apps were higher in psychopathy (slightly) and sexual compulsivity. Next, the responsibility for condom use, contraception, and alcohol on dates was tested.

*Condom use and contraception.* Two series of 2 (single/partnered) × 2 (dating app use) × 2 ANOVAs (A: condom use on a date; B: contraception responsibility) were run with traits and sexual satisfaction as dependent variables for both men and women. No differences in men (*F*[6, 268] = 1.11, *p* = 0.36, *η_p_*^2^ = 0.02) or women (*F*[6, 185] = 0.42, *p* = 0.86, *η_p_*^2^ = 0.01) were observed regarding their position on condom use. Women using dating apps who were pro condom use were slightly more narcissistic than those who were against their use, with no difference among women not using dating apps (*F*[6,2 32] = 5.30, *p* = 0.02, *η_p_*^2^ = 0.04). Women favoring condom use on hook-ups were more Machiavellian than those who did not, regardless of their dating app use (*F*[6, 141] = 11.16, *p* < 0.01, *η_p_*^2^ = 0.07). Regarding contraception, no interactions were observed in men (*F*[6, 269] = 0.49, *p* = 0.82, *η_p_*^2^ = 0.01), nor in women (*F*[6, 184] = 0.92, *p* = 0.48, *η_p_*^2^ = 0.03). Men who felt responsible for contraception were slightly less sexually compulsive but slightly more satisfied with their own and their partner’s sexual activity (*F*[2, 273] = 6.38, *p* = 0.01, *η_p_*^2^ = 0.01); they were also slightly less psychopathic when not using dating apps. There was no difference in psychopathy among those men who did not consider themselves responsible (*F*[2, 280] = 4.30, *p* = 0.04, *η_p_*^2^ = 0.04). Single men who felt responsible for contraception were slightly less satisfied with their intimate partners’ sexual activities compared to those men who did not, with no difference among men in a committed relationship (*F*[2, 280] = 5.01, *p* = 0.03, *η_p_*^2^ = 0.02). Similarly, single men not using Tinder feeling responsible for contraception were slightly less satisfied with their own sexual potential than men who did not, whereas no difference emerged among dating app users (*F*[2,280] = 4.23, *p* = 0.04, *η_p_*^2^ = 0.02).

*Alcohol on dates*. A last series of 2 (single vs. non-single) x 2 (alcohol on a date: yes/no) ANOVAs was run with traits and sexual satisfaction as dependent variables for men and women. No interactions were observed in men (*F*[6, 269] = 0.49, *p* = 0.82, *η_p_*^2^ = 0.01), nor in women (*F*[6, 188] = 0.70, *p* = 0.81, *η_p_*^2^ = 0.02). Men who considered drinking on a date were slightly more psychopathic than men not considering drinking (*F*[2, 273] = 3.23, *p* = 0.04, *η_p_*^2^ = 0.05). Women who considered alcohol to be part of a date were slightly more narcissistic than those who did not (*F*[3, 191] = 3.35, *p* = 0.02, *η_p_*^2^ = 0.05).

## Discussion

In this study, single and partnered individuals using dating apps were investigated regarding self-reported sex organized via dating apps (i.e., Tinder sex), risky sexual behaviors, personality, and sexual satisfaction with oneself and one’s current partner’s sexual performance. Risky sexual behaviors examined covered condom use, contraceptive responsibility, and alcohol consumption on dates. Exploring sexual infidelity (March et al., 2024) and health risks (Flesia et al., 2021) of dating app users expands knowledge of sexual strategies in modern environments and provides insights beyond intentions of app use (Lyons et al., 2022), such as cheating. Personality was measured via traits associated with casual sex (i.e., narcissism, Machiavellianism, psychopathy, and sexual compulsivity). Based on frequently observed sex differences (March et al., 2024), men and women were investigated separately. Notably, non-heterosexuals can make up to 23% of users (Barrada and Castro, 2020), yet gays, lesbians, and bisexuals report similar partner preferences such as heterosexuals (Klümper et al., 2024; Lucas et al., 2011). Thus, the focus here lies on communalities among dating app users, not differences in sexual orientation, and on investigating sex differences as individual differences in preferences seem to be rooted in biological sex (Kostic and Scofield, 2022).

Labeling dating app users as less trustworthy (Silva et al., 2019) was confirmed as 52% of users who arranged dates via apps also reported Tinder-sex, and partnered users reported more Tinder-sex than single users across both sexes. Further, male and female dating app users were more opportunistic (i.e., higher in psychopathy and sexual compulsivity) than non-users, and particularly Machiavellians reported more Tinder-sex. Men in general and women in relationships reported extended levels of Machiavellianism. Partnered women who had Tinder-sex were more psychopathic than single women who had Tinder-sex (supporting H1). However, sexual compulsivity was slightly higher in male but not in female individuals who had Tinder-sex. These findings might imply two reasons. One, a more strategic approach to the sex of women in general (e.g., Haselton and Buss, 2000), as more differentiated effects of Machiavellianism (i.e., tactics and agentic goal setting) and psychopathy (i.e., impulsivity) were observed in women. Two, psychopathic traits in women engaging in dating app use and Tinder-sex might indicate a short-term, opportunistic orientation linked to borderline characteristics (Sprague et al., 2012). This might reflect unstable relationship patterns of borderline characteristics (Brüne, 2016), as women engaging in Tinder-sex are more satisfied with their own and their partner’s sexual performance.

Beyond intersexual differences (March et al., 2024), corresponding intrasexual differences regarding risky sexual behaviors were expected. Men’s sexual behaviors and attitudes hardly differed regarding their relationship status, personality, or sexual satisfaction. Women, in contrast, were more strategic: Machiavellian dating app users were more likely to forgo condoms during sex, whereas non-users were more likely to use condoms. This could be an indication of a deeply rooted reproductive drive evident among women in contexts of uncommitted sex (Simpson and Belsky, 2008). Psychopathic men and narcissistic women are more likely to drink alcohol on dates. This suggests opportunistic, risk-associated behaviors in male drinkers, while narcissistic women may enhance their first impression by accepting drinks. As Machiavellian women prioritize harm avoidance more than men (Czibor et al., 2017), some of these behaviors can be risk-managing tactics during hook-ups and casual sex to Haselton and Buss (2000). This extends beyond viewing risk-taking as mere reckless opportunism by psychopaths, indicating that even among dating app users and those prone to uncommitted sex, women adopt more tactical approaches to risky sexual behaviors than men (partially supporting H2).

These findings align with international results (Australia, Greece, Malaysia; Alavi et al., 2018; Apostolou, 2024; March et al., 2024) that link infidelity to personality (i.e., Machiavellianism and psychopathy) rather than cultural differences. The same pattern emerged for the drinking-Tinder-sex link, confirming prior research (Lopez et al., 2023) showing no overall sex differences, but personality-based differences. Regarding sexual health, contraception responsibility was confined to individuals in committed relationships regardless of their sex, and reduced condom motivation among short-term oriented individuals suggests lifestyle and personality outweigh economic and cultural factors in public health concerns about sexual protection behaviors––consistent with STI prevalence patterns (Zheng et al., 2022).

This study expands and specifies strings of research on sexual infidelity, risky sex, and sexual strategies in the new environment of dating apps. Different sexual strategies, such as an opportunistic approach to sex for men and the more tactical behaviors of women, were replicated. Furthermore, each Dark Triad trait was linked to distinct behaviors, aligning with international research. Yet, despite sexual compulsivity being higher among all dating app users, mainly men showed associated behaviors such as Tinder-sex and less responsibility for contraception. Sexual infidelity and risky sex behaviors were observed, but mostly limited to each sex itself (H2), sex-specific to potential consequences (Haselton and Buss, 2000), or context-dependent (i.e., responsibility in committed relationships). One pattern explaining women’s additional behaviors and attitudes may be the female manifestation of psychopathy as a sexual strategy—namely, borderline. Moreover, opportunistic cheating could provide access to good genes. These findings go beyond intentions and give a first impression of dating app users’ sexual behaviors and their link to personality.

## Limitations

This study used a cross-sectional and quasi-experimental design, which inhibits causal interpretations. WEIRD data and focusing on apps with mostly heterosexual users limit the findings’ generalizability, as sexual minorities were not explicitly investigated, but included in the sample. The trait-level assessment of psychopathy did not capture the impulsivity facet. This might be of interest, particularly regarding sexual compulsivity and potential interactions around the behavior of hypersexual, borderline women (Carvalho et al., 2015). A larger sample would allow sufficient subgroup sizes and extreme group analyses. One element not captured was users’ and their mates’ mate values. This might be important, as a rare opportunity for high(er) valued hook-up might increase the chance of sex, just as self-perceived mate value increases the likelihood of cheating (Alexopoulos et al., 2020), and mismatches in mate value might lead individuals to look for other partners. Unconsidered were planful cheaters who could meet other partnered individuals, particularly for extradyadic sex. Future research on dating apps should explore agentic behavior in different relationships, considering the little sanctioning that is expected online.

## Conclusion

This study explored several still-open questions regarding the sex-related risk behaviors and attitudes of dating app users, and their associations with personality. Sexual infidelity was reported among sexually compulsive men but not among women. Men having Tinder-sex displayed even higher levels of personality characteristics associated with uncommitted sex, independent of their relationship status or sexual satisfaction. Women cheating via dating apps were sexually more satisfied but less likely to use condoms. While male users of dating apps mainly displayed an opportunistic approach, women using dating apps showed different and more complex patterns of sexual strategies. Results align with intercultural research.

## Data Availability

The datasets presented in this study can be found in online repositories. The names of the repository/repositories and accession number(s) can be found in the article/supplementary material.
